# Difference in orientation of the talar articular facets between healthy ankle joints and ankle joints with chronic instability

**DOI:** 10.1002/jor.25068

**Published:** 2021-05-12

**Authors:** Roeland P. Kleipool, Sjoerd A. S. Stufkens, Jari Dahmen, Gwendolyn Vuurberg, Geert J. Streekstra, Johannes G. G. Dobbe, Leendert Blankevoort, Markus Knupp

**Affiliations:** ^1^ Department of Medical Biology, Amsterdam UMC University of Amsterdam, Amsterdam Movement Sciences Amsterdam The Netherlands; ^2^ Academic Center for Evidence‐Based Sports Medicine (ACES) Amsterdam The Netherlands; ^3^ Department of Orthopedic Surgery, Amsterdam UMC University of Amsterdam, Amsterdam Movement Sciences Amsterdam The Netherlands; ^4^ Amsterdam Collaboration on Health & Safety in Sports (ACHSS), AMC/VUmc IOC Research Center Amsterdam The Netherlands; ^5^ Department of Radiology and Nuclear medicine, Amsterdam UMC University of Amsterdam, Amsterdam Movement Sciences Amsterdam The Netherlands; ^6^ Department of Radiology and Nuclear medicine Rijnstate Ziekenhuis Arnhem The Netherlands; ^7^ Department of Biomedical Engineering and Physics, Amsterdam UMC University of Amsterdam, Amsterdam Movement Sciences Amsterdam The Netherlands; ^8^ Mein Fusszentrum, Basel University of Basel Switzerland

**Keywords:** ankle joint, subtalar joint, anatomy, chronic ankle instability, computed tomography

## Abstract

Since both the talocrural and subtalar joints can be involved in chronic ankle instability, the present study assessed the talar morphology as this bone is the key player between both joint levels. The 3D orientation and curvature of the superior and the posteroinferior facet between subjects with chronic ankle instability and healthy controls were compared. Hereto, the talus was segmented in the computed tomography images of a control group and a chronic ankle instability group, after which they were reconstructed to 3D surface models. A cylinder was fitted to the subchondral articulating surfaces. The axis of a cylinder represented the facet orientation, which was expressed by an inclination and deviation angle in a coordinate system based on the cylinder of the superior talar facet and the geometric principal axes of the subject's talus. The curvature of the surface was expressed as the radius of the cylinder. The results demonstrated no significant differences in the radius or deviation angle. However, the inclination angle of the posteroinferior talar facet was significantly more plantarly orientated (by 3.5°) in the chronic instability group (14.7 ± 3.1°) compared to the control group (11.2 ± 4.9°) (*p* < 0.05). In the coronal plane this corresponds to a valgus orientation of the posteroinferior talar facet relative to the talar dome. In conclusion, a more plantarly and valgus orientated posteroinferior talar facet may be associated to chronic ankle instability.

## Graphical abstract

To investigate talar morphological differences between healthy controls and patients with chronic ankle instability (CAI) we modeled the superior and posteroinferior talar facets as cylinders. In relation to the superior facet the posteroinferior facet directed more plantarly in the CAI group (14.7 ± 3.1°) versus the controls (11.2 ± 4.9°) (*p* < 0.05). The posteroinferior talar facet stands in slight valgus relative to the superior talar facet. The talar morphology may be a risk factor for CAI.

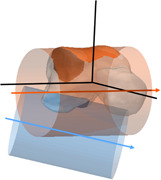

## 1 INTRODUCTION

Identifying factors that are related to chronic ankle instability (CAI) can decrease unnecessary delay in diagnosis and treatment, increase the effectiveness of treatment as well as reduce the direct costs of medical care, and decrease the high socioeconomic burden associated with ankle sprains.^1^ CAI is defined as instability of the ankle joint with the sense of giving way, episodes of recurrent ankle sprains, with or without the presence of joint laxity.^2^, ^3^ Varus malalignment of the lower leg or the hindfoot has shown to increase the risk of CAI.^4^, ^5^, ^6^, ^7^, ^8^, ^9^, ^10^, ^11^ The malalignment of the hindfoot is radiographically defined as the calcaneal offset in relation to the longitudinal axis of the tibia. A varus malalignment shifts the axial load laterally compared to a neutral alignment, which subsequently creates an inversion moment that can result in a lateral ankle sprain if not timely countered at foot landing.^12^ While the correlation of tibial malalignment and ankle joint instability has been extensively analyzed,^5^ only few studies assess the importance of hindfoot varus alignment on the development of CAI.

The alignment of the hindfoot is influenced by the shape of the bones. In particular, the orientation of the articulating surfaces as well as the deformation in the mid‐ and forefoot induced by foot loading play an important role in this alignment. Both the talocrural and subtalar joints can be involved in CAI.^2^ The talus is a key player between both joint levels and therefore also regarding the alignment of the hindfoot. Malalignment can be present at any level in the mechanical chain from tibia to the ground.^7^, ^13^ The influence of the morphology of the talocrural joint on the development of CAI is well documented. For example, the radius of the talar dome was shown to be larger in patients with CAI than in controls.^14^ However, controversies exist on the role of the morphology of the subtalar joint in the relationship with CAI. Furthermore, abnormal orientation at one joint level in the mechanical chain of the hindfoot can be compensated by an adaptation at another joint level.^7^, ^14^ This makes it important to determine the morphology per joint level and the interrelationships in orientation at these joint levels.

A three‐dimensional (3D) analysis of the orientation of the articulating facets in the mechanical chain of the hindfoot is currently missing in present literature. Previously, the determination of subtalar joint orientation was reported in a two‐dimensional (2D) manner (e.g.,^4^, ^13^, ^15^, ^16^), however, this is prone to errors and has limitations.^9^, ^17^, ^18^

As a result, the present study was designed to address the potentially present differences in the morphology of the talar facets between subjects with CAI and those without (i.e., healthy controls). A comparison was made between healthy controls and patients with CAI to assess the differences in orientation of the posteroinferior subtalar joint relative to the talar dome in 3D space, and of the curvature of these facets. The hypothesis is that this orientation and curvature is significantly different between patients with CAI and healthy controls.

## 2 MATERIALS AND METHODS

### 2.1 Datasets

For this retrospective case‐controlled study, three datasets from three previous studies were used.^18^, ^19^, ^20^ Each study was approved by the local Institutional Review Board. In all three studies, the distal tibia, talus, and calcaneus of the participants were imaged in supine position with computed tomography (CT) (0.3 <voxel size <0.4557 mm, tube charge of 120 kV, and radiation dose of 150–160 mAs, *Brilliance 64 CT scanner, Philips Healthcare, Amsterdam, The Netherlands*). A total of 40 ankles from two groups of 20 healthy volunteers each with non‐symptomatic feet/ankles were included to form the control group for the present study.^18^, ^19^ In case both sides were scanned, an arbitrary choice was made for one of the sides assuming that the differences within a subject is smaller than the differences between subjects.^18^ The CAI group included 12 patients with CAI (Table 1).^20^ If both sides of the patient were affected, the side with the highest incidence of previous ankle sprains was selected. No a‐priori power analysis was performed since there were no data available to perform such analysis.

**Table 1:** Participant characteristics of the control group and group of patients with chronic ankle instability (CAI)

**Group**	**Number of tali**	**Number of male/female**	**Mean age (years)**	**Standard deviation (years)**	**Minimal age (years)**	**Maximal age (years)**	**Range (years)**
Control^18^, ^19^	40	20/20	31.1	9.5	22	59	37
CAI^20^	12	8/4	27.3	10.9	19	59	40

### 2.2 Hanalysis

The talus was segmented from the 3D‐CT image and modeled as 3D polygons using custom made software,^21^ which was developed and validated at our academic hospital.^22^ The following subchondral articular joint surfaces of the talus were selected using the selection tool in Blender (software version 2.79, GNU General Public License): the talar superior facet (the facet of the talus that articulates with the tibial plafond, also known as the talar dome), and the talar posteroinferior facet (the facet that articulates with the posterior facet of the calcaneus). Selection of these surfaces was done by one researcher (RPK, who has more than five years of experience in segmentations and 3D modeling of the hindfoot). No blinding for study group was performed for a possible bias, since the observer had no overview of the end results. In the first step, segmentation was performed. Secondly, the selection of the surface of the talar dome was performed. Finally, the selection of the posterior subtalar surface was performed without the presence of images of the talar dome. Subsequent analysis was performed by computer algorithms not requiring any user intervention.

### 2.3 Data processing

**Figure 1:**
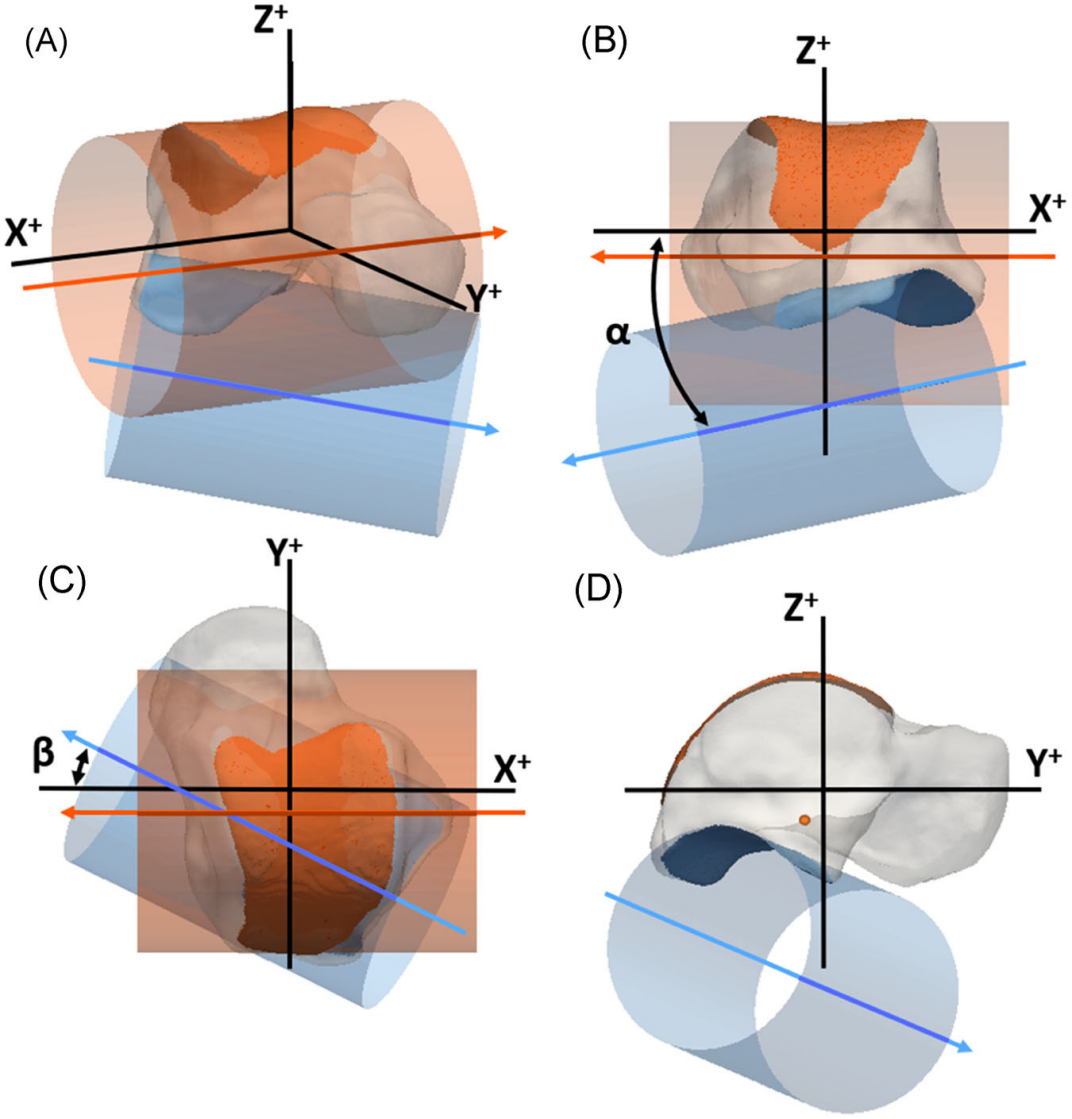
Graphical representation of the talus (transparent), the superior talar facet (orange, TalusSF), and posteroinferior talar facet (blue, TalusIF) in (A) anterolateral view; (B) posterior view; (C) superior view; (D) lateral view. The facets were modeled as (segments of) cylinders (transparent in matching colors) by fitting these cylinders to the articulating surfaces. The arrows (in matching color) represent the direction of the axes of the cylinders. The orientation of the TalusIF is represented by the orientation of its cylinder axis in a local coordinate system, and is expressed by an inclination angle (*α* in B) and a deviation angle (*β* in C) (see text for further details). The positive *X*‐axis is directed laterally, the positive *Y*‐axis is directed anteriorly, and the positive *Z*‐axis is directed proximally [Color figure can be viewed at wileyonlinelibrary.com]

The facets were modeled as a segment of a cylinder.^18^ Hereto, a cylinder was fit to the points in each selected polygon surface using a nonlinear least‐squares optimization process (Figure 1). The cylinders were named TalusSF and TalusIF, for the superior and posteroinferior talar facet, respectively. The radius of the cylinder represents the curvature of the facet. To quantify to what extent the facet matches with the cylindrical shape, the root mean square error between the points of the facet and cylinder was calculated. A large error indicates that the articulating surface does not fit well to the cylinder, for example because the surface is flat. On the other hand, a small error indicates a good match with a cylindrical surface.

Another outcome of the present study was the interrelationship between the two facets of the talus. The orientation of TalusIF in respect to TalusSF describes (a part of) the talar morphology. To align all tali uniformly and reproducibly, a local orthogonal right‐handed coordinate system was defined with the following steps. All left tali were mirrored to right tali for further analyses in a corresponding orientation. The *X*‐axis was set parallel with the cylinder's axis of TalusSF and was in a medial‐lateral direction. Next, the geometric principal axes of the subject's talus were determined. The *Y*‐axis was defined using the Gram‐Schmidt process with the determined *X*‐axis and the talar principal axis in antero‐posterior direction.^23^ The *Z*‐axis was defined perpendicular to the *X*‐ and *Y*‐axis with the positive direction orientated superiorly. The orientation of a cylinder was defined by a direction vector, which was chosen in approximately the medial direction in this study. From this, it follows that the direction vector of TalusSF was parallel to the *X*‐axis, pointing in the negative direction.

The orientation of TalusIF was defined by two angles (Figure 1B,C). The inclination (*α*) angle was determined between the direction vector and the *XY* plane, and the deviation (*β*) angle was determined between the negative *X*‐axis and the projection of the direction vector in the *XY* plane. Cylinder fittings and determinations of the angles were processed with custom made routines in Matlab (MATLAB Release 2019; The MathWorks, Inc.).

### 2.4 Intraobserver and interobserver analysis

The intraobserver reliability in cylinder orientation and radius, introduced by manual selection of the facet surfaces, was evaluated using three randomly chosen feet; one per study cohort. Surface selection was done three times with at least 1 week in between. Interobserver reliability was evaluated based on manual selection of the facet surfaces by two observers (RPK and JD; both researchers have several years of experience in segmentation and 3D software) of nine randomly selected hindfeet (three from the CAI group and six from the control group). The two observers were blinded to each other's selections.

### 2.5 Statistical analyses

The orientation angles of TalusIF, the radii of the cylindrical fits, and the root mean square errors were analyzed for normal distribution with the Shapiro–Wilk test. For normally distributed data, outliers were identified as exceeding the value of three times the standard deviation from the mean. For non‐normally distributed data, outliers were identified as exceeding the value of 1.5 times the interquartile range from the median. An outlier in one of the orientation angles was removed from further analyses for both angles. An outlier in the root mean square error or radius of a cylinder facet was removed from further analyses for that facet. The differences between the control group and the CAI group were tested with an independent Student *t* test in case of normally distributed data and with a Mann–Whitney *U* test for non‐normally distributed data. The Hegdes' *g* value for effect size was calculated. The Hedges' *g* value was interpreted as small effect (closest to 0.2); medium effect (closest to 0.5); or large effect (closest to 0.8).^24^

The intraclass correlation coefficient (ICC) was calculated as a measure of intraobserver and interobserver reliability. The ICC was interpreted as poor (≤0.40); moderate (0.40−0.75); substantial (0.75 – 0.90); or excellent reliability (>0.90).^25^

The statistical analyses were performed with SPSS (IBM SPSS Statistics for Windows, Version 26.0. Released 2019; IBM Corp.). A significance level of 0.05 was used for all tests. Data are presented as mean ± 1 *SD* for normally distributed data and as median (interquartile range) for non‐normally distributed data.

## 3 RESULTS

### 3.1 Facet orientation

**Figure 2:**
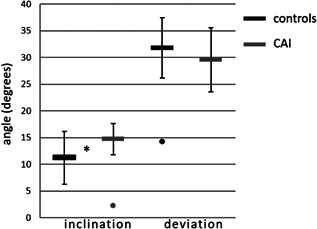
Inclination and deviation angle (mean and standard deviation) of the posteroinferior talar facet (TalusIF) of the control group and the chronic ankle instability group (CAI). The dots indicate outliers (>3 times the standard deviation from the mean value). The asterisk indicates a statistical significant difference between the two groups (*p* = 0.008)

The orientation angles were normally distributed. One outlier was identified in the inclination angle in the CAI group, and one outlier was identified in the deviation angle in the control group. The orientation of TalusIF was in an antero‐medial‐plantar direction. The inclination angle of TalusIF was statistically significantly more plantarly orientated in the CAI group (14.7 ± 3.1°, *n* = 11) compared to the control group (11.2 ± 4.9°, *n* = 39), with a large effect size, *t*(26) = −2.85, *p* = 0.008, *g* = 0.76, with a mean difference of 3.5° (Figure 2). Levene's test indicated unequal variances (*F* = 4.3, *p* = 0.043), that is, degrees of freedom were adjusted from 48 to 26. The deviation angle of TalusIF was not statistically significantly different between the CAI (29.5 ± 6.3°, *n* = 11) and the control group (31.8 ± 5.7, *n* = 39), *t*(48) = 1.12, *p* = 0.263.

### 3.2 Cylinder radius

**Figure 3:**
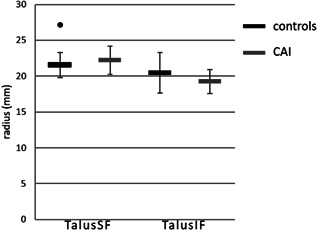
Cylinder radii (mean and standard deviation) of the superior talar facet (TalusSF) and the posteroinferior talar facet (TalusIF). The dot indicates an outliers (>3 times the standard deviation from the mean value). CAI, chronic ankle instability

The cylinder radii were normally distributed. One outlier was identified for TalusSF in the control group, and one outlier in the CAI group for the root mean square error. The cylinder radii of TalusSF were not statistically significantly different between the CAI (22.2 ± 2.2 mm, *n* = 11) and the control group (21.5 ± 1.8 mm, *n* = 39), *t*(48) = −1.06, *p* = 0.249 (Figure 3). In the root mean square error of TalusIF of the CAI group one outlier was identified, and one in the control group. Also, for the cylinder radii of TalusIF, no statistical significant differences were demonstrated between the CAI group (19.2 ± 1.8 mm, *n* = 11) and the control group (20.5 ± 2.9 mm, *n* = 39), *t*(26) = 1.40, *p* = 0.084. For TalusIF, Levene's test indicated unequal variances (*F* = 4.3, *p* = 0.044). Therefore, degrees of freedom were adjusted from 50 to 26.

### 3.3 Root mean square errors of the fits

The root mean square errors of the cylinder fit were normally distributed, except for TalusIF in the CAI group. After removal of this outlier, the remaining data were normally distributed. The root mean square errors of TalusSF were not statistically significantly different between the CAI (0.45 ± 0.09 mm, *n* = 11) and the control group (0.41 ± 0.09, *n* = 39), *t*(48 = −1.14, *p* = 0.259. The root mean square errors of TalusIF were not statistically significantly different between the CAI (0.39 ± 0.06 mm, *n* = 11) and the control group (0.43 ± 0.11, *n* = 39), *t*(48) = 1.22, *p* = 0.228.

### 3.4 Intraobserver and interobserver analyses

The intra‐ and interobserver reliability showed excellent reliability for each of the outcome measurements (Table 2). The differences in the intra‐observer measurements were smaller than the interobserver measurements. The largest differences were found in deviation angle of TalusIF.

**Table 2:** Intraclass correlations coefficients (ICC) and the 95% confidence interval for the intraobserver analysis and interobserver measurement

	**Intraobserver (*n* = 9)**			**Interobserver (*n* = 9)**		
**Outcome measure**	**ICC**	**95% Confidence interval**	**Absolute difference**	**ICC**	**95% Confidence interval**	**Absolute difference**
Inclination angle TalusIF	0.994	0.944–1	0.46 ± 0.39 (0.05‐1.28)	0.989	0.953–0.998	0.71 ± 0.85 (0.04‐2.18)
Deviation angle TalusIF	0.981	0.821–1	0.48 ± 0.36 (0.05‐1.03)	0.988	0.949–0.997	1.27 ± 1.07 (0.24‐3.08)
Radius TalusSF	0.969	0.792–0.999	0.08 ± 0.05 (0.03‐0.17)	0.977	0.828–0.990	0.29 ± 0.19 (0.03‐0.59)
Radius TalusIF	0.994	0.952–1	0.25 ± 0.17 (0.00‐0.59)	0.985	0.932–0.996	0.49 ± 0.36 (0.06‐1.12)

*Note*: Also, the average ± 1 *SD* and range (minimal–maximal) of the absolute differences are presented for the intra‐ and inter‐observations. TalusSF: cylinder of superior facet of the talus. TalusIF: cylinder of posteroinferior facet of the talus. Absolute differences angles in degrees, and radius in mm.

## 4 DISCUSSION

The present study investigated the orientation and curvature of the superior and posteroinferior talar articulating surfaces of nonsymptomatic individuals and of patients with chronic ankle instability. Previous reports have identified a varus hindfoot deformity to be a risk factor in the development of CAI after a first ankle sprain.^4^, ^5^, ^6^, ^7^, ^8^, ^9^, ^10^, ^11^ Since both the talocrural and subtalar level can be involved in CAI,^2^ the present study assessed the talar morphology as this bone is the key player between both joint levels.

**Figure 4:**
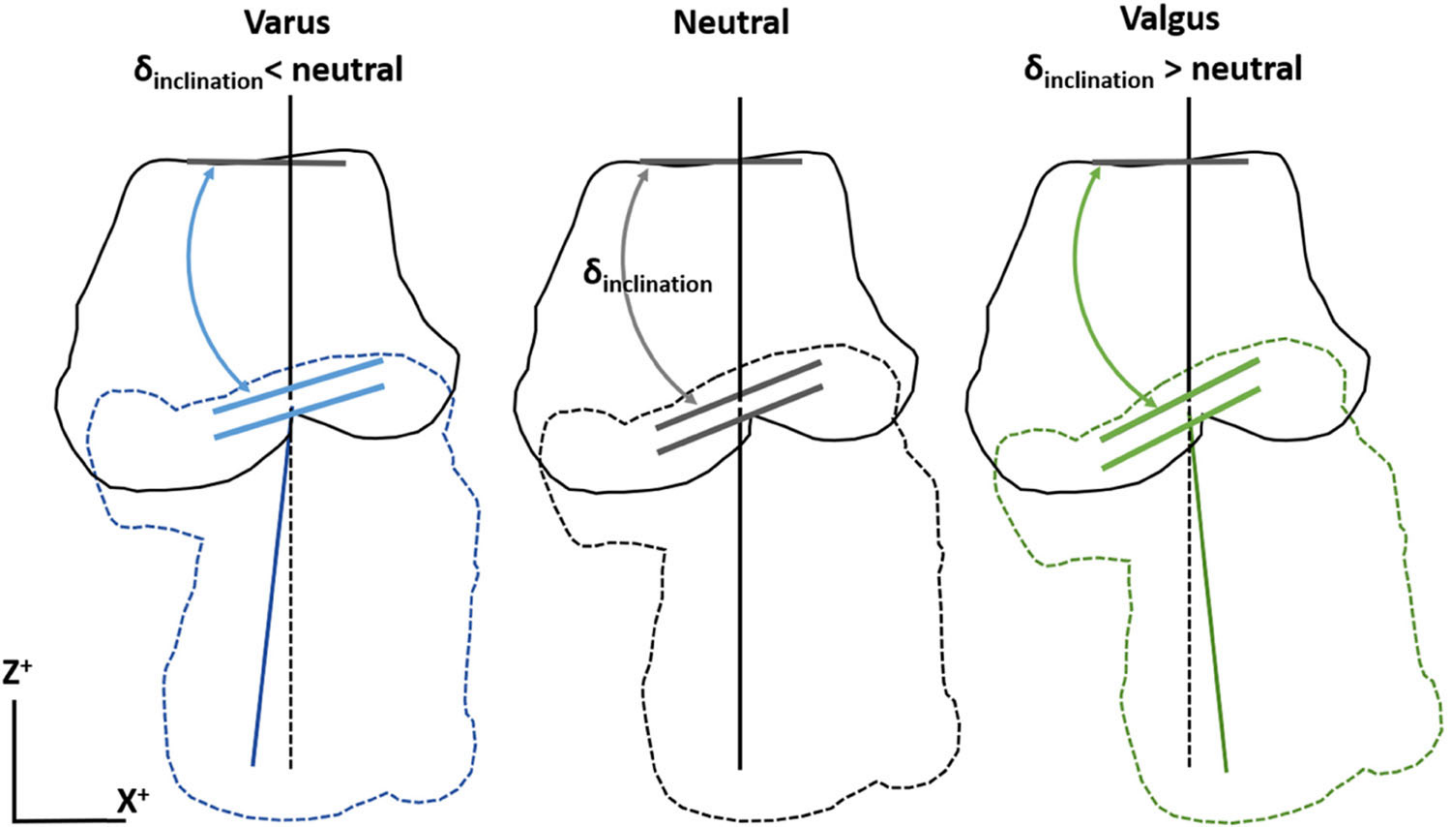
Schematic contour drawing of the talus and calcaneus from a posterior view (same view as in Figure 1B) for three hypothetical inclination angles of the posteroinferior talar facet. In each alignment the orientation of three facets is represented by three short lines: the upper for the superior talar facet, the middle for the posteroinferior talar facet, and the lower for the posterior calcaneal facet. The superior talar orientation is identical between the three alignments. The lower two are parallel and different between the three alignments. The upper and lower vertical lines represent the coronal alignment of the talus and calcaneus, respectively. A larger inclination angle of the posteroinferior talar facet places the calcaneus in a valgus position relative to the talus, and a lower inclination angle places the calcaneus in a varus position [Color figure can be viewed at wileyonlinelibrary.com]

The most important finding was that the mean inclination angle of the cylinder's axis of the posteroinferior talar facet was statistically significantly more plantarly orientated, by 3.5 degrees, in the CAI group compared to the control group. In a coronal plane this corresponds to a talus with a slight valgus of the posteroinferior facet relative to the talar dome (Figure 4).

We expected to find a varus orientation of the subtalar joint in patients with CAI when comparing them to the healthy population. This would have been in accordance with earlier studies who found a correlation between varus malalignment and CAI.^4^, ^5^, ^9^ In our cohort, however, patients with CAI were found to have a subtalar joint in a slight valgus orientation. These findings are in line with a recent hypothesis from Tümer et al., who found that the calcaneus in patients with CAI creates a higher valgus‐directed moment due to particular calcaneal bony shapes present in these patients.^12^ Patients with CAI may reduce the valgus‐directed moment and an unwanted medial shift of the ground reaction force by actively creating a varus‐directed moment, that results in a lateral ankle sprain if not timely countered at foot landing. Our results indicate that the posteroinferior talar facet places the calcaneus in valgus position relative to the talus, and this could also be actively compensated by a varus‐directed moment.^7^, ^13^ The clinical impact of these finding remains uncertain. The literature on CAI in patients with tibia or hindfoot varus alignment shows that they benefit from realignment osteotomies to avoid ligament reconstruction failure caused by the mechanical malalignment.^26^, ^27^ Corrections of any malalignment, however, should be planned at the level of the center of rotation of angulation. The offset of the calcaneus in relation to the tibia in ankle malalignment can surgically be corrected with a corrective osteotomy of the distal tibia or the calcaneus and with a corrective subtalar joint fusion. Knowledge of the orientation of the ankle and the subtalar joint in the preoperative planning therefore may improve the surgical outcome. Also, in planning a joint replacement, a sound knowledge of the joint morphology is needed with consideration of the interrelationship of both joint levels.^28^

No statistically significant differences were demonstrated between the groups for the radii nor root mean square errors of the cylinder fits. The root mean square error indicates how well the facets fit to a cylinder. These results imply that the morphology of the two facets was comparable between the two study groups. Frigg et al.^14^ demonstrated an inverse relationship between the curvature of the talar dome and the stability of the ankle joint. This larger curvature alone cannot explain a more instable joint configuration. The coverage of the tibial surface is also important. The most instable joint configuration would be a combination of a small tibial coverage and a large curvature.^14^ We did not examine the tibial coverage. In both our groups, the average radius of the talar dome was larger compared to the corresponding group of Frigg et al. The most comparable values were found in the CAI group (control group 21.5 ± 1.8 mm and 17.7 ± 1.9 mm, and CAI group 22.2 ± 2.1 mm and 21.2 ± 2.4 mm for our study and Frigg et al. respectively). Our 3D surface fit possibly results in a larger radius than the 2D radiographically determined radius.^14^ Another explanation is that in our study groups we had a larger percentage of male subject that resulted in the larger average radius.

The present study focused on the bony morphology of the talus as a possible risk factor for CAI. Caution is required when interpreting the results of this one factor, namely the bony morphology, when in fact CAI is multi‐factorial.^2^ There is an overlap in individuals with and without CAI (Figures 2 and 3). Subjects that served as controls may in the future acquire CAI. These could be the individuals that also have slightly more valgus in the talus, but that does not have to be the case. Similarly, there were patients in our CAI group who did not have this slight valgus in the talus. Other causes for developing CAI were probably of influence.

It is important to realize that the overall hindfoot alignment could still be varus, but that in our analysis of an isolated talus at the joint levels this could not be demonstrated. The patients could have an overall hindfoot varus alignment when the medial distal tibial angle^29^ is more in varus than the subtalar joint is in valgus. However, we did not assess the relationship of the superior facet of the talus with the longitudinal axis of the tibia or other measures for the alignment. Other reasons could come from differences in 3D versus 2D measurements. Previous reports on the orientation of the two joint levels used coronal planes of CT imaging.^4^, ^16^ However, the posteroinferior talar surface is located more posteriorly than the superior talar surface. Because of this, a coronal plane at for example the center of the superior facet crosses the posteroinferior facet at the anterior edges.^16^, ^30^ Furthermore, because of the oblique orientation in all anatomical planes of the posterior subtalar joint, the angle of the joint is dependent on the chosen antero‐posterior location of the coronal plane.^18^ These observations of the 3D morphology need further exploration and raise the question whether the 2D analyses are adequate to grasp the complex 3D morphology.

This study has several limitations other than not assessing the hindfoot alignment. The true articulating cartilaginous surfaces cannot be seen in CT images, and as such only the subchondral surfaces were analyzed. Nonhomogeneous thickness distribution of the cartilage may shift the cylinder. This could be addressed in a study that uses Magnetic Resonance Imaging. Further, only 12 CAI patients were included. After removal statistical outliers, this was further reduced. Future research could include more patients with CAI.

Up to today, the mechanics of the subtalar joint are not fully elucidated. Different in vivo, ex vivo, and modeling studies have given more insight into the kinematics of the subtalar joints.^28^ How our cylinder models reflect in the kinematics of the hindfoot is not clear. The axis of rotation of the subtalar joint does not correspond with what can be predicted from our cylinder model of the posterior subtalar joint.^28^ The subtalar joints comprise of more than only the posterior articulating surfaces. The whole subtalar joint is more complex. Our method could be used in determining the 3D orientations of the hindfoot bones, and does not reflect the full complex subtalar joint morphology.

The strength of this present study was that the interrelationship of the superior and posteroinferior talar facets could be determined in 3D without influence of the position of the foot in the CT scanner. The applied method for selecting the articular surface on 3D surface models proved to be very reliable based on the results of the intraobserver and interobserver analyses, but was laborious. A semi‐automatic procedure can make this step more efficient in the future.

The present study provides a next step in identifying morphology of bony structures in the hindfoot being associated with CAI. It is the first study that analyzed the interrelationship of the talocrural and subtalar joints in 3D. Future studies should focus on a method to determine the orientation at each joint level in the chain from the knee to the ground in 3D with a golden standard for a frame of reference, preferably in a weight bearing condition.

In conclusion, it was found that the morphology of the talus itself does not necessarily need to be in “varus” to contribute to CAI. It was demonstrated that CAI patients have a more valgus position of the posteroinferior talar facet that may be a risk factor for CAI by varus compensation. The relationship between the superior and posteroinferior facets of the talus in relation to the lower leg axis in varus and valgus deformities, the overall alignment, needs further research.

## AUTHOR CONTRIBUTIONS

All authors contributed to the study design. Roeland P. Kleipool, Jari Dahmen, Johannes GG. Dobbe, Geert J. Streekstra, and Leendert Blankevoort contributed to the data analysis, and Roeland P. Kleipool and Jari Dahmen contributed to the interobserver analysis. Roeland P. Kleipool, Sjoerd AS. Stufkens, Jari Dahmen, Gwendolyn Vuurberg, and Markus Knupp participated in interpretation of data and clinical significance. Roeland P. Kleipool drafted the manuscript, which was critically revised and approved by all authors.

## References

[1] Vuurberg G , Hoorntje A , Wink LM , et al. Diagnosis, treatment and prevention of ankle sprains: update of an evidence‐based clinical guideline. Br J Sports Med. 2018;52(15):956. 29514819 10.1136/bjsports-2017-098106

[2] Kobayashi T , Gamada K . Lateral ankle sprain and chronic ankle instability: a critical review. Vol. 7, Foot & ankle specialist. United States; 2014. p. 298–326. 10.1177/1938640014539813 24962695

[3] Hertel J . Functional Anatomy, Pathomechanics, and Pathophysiology of Lateral Ankle Instability. J Athl Train. 2002;37(4):364‐375. 12937557 PMC164367

[4] Van Bergeyk AB , Younger A , Carson B . CT analysis of hindfoot alignment in chronic lateral ankle instability. Foot Ankle Int. 2002;23(1):37‐42. 11822690 10.1177/107110070202300107

[5] Strauss JE , Forsberg JA , Lippert FG, 3rd . Chronic lateral ankle instability and associated conditions: a rationale for treatment. Foot Ankle Int. 2007;28(10):1041‐1044. 17923051 10.3113/FAI.2007.1041

[6] Bonnel F , Teissier P , Maestro M , Ferré B , Toullec E , AFCP . Biometry of bone components in the talonavicular joint: a cadaver study. Orthop Traumatol Surg Res. 2011;97(6 Suppl):S66‐S73. 21807576 10.1016/j.otsr.2011.06.005

[7] Sangeorzan A , Sangeorzan B . Subtalar Joint Biomechanics: From Normal to Pathologic. Foot Ankle Clin. 2018;23(3):341‐352. 30097078 10.1016/j.fcl.2018.04.002

[8] Vuurberg G , Wink LM , Blankevoort L , et al. A risk assessment model for chronic ankle instability: indications for early surgical treatment? An observational prospective cohort ‐ study protocol. BMC Musculoskelet Disord. 2018;19(1):225. 30021553 10.1186/s12891-018-2124-5 PMC6052530

[9] Lintz F , Bernasconi A , Baschet L , Fernando C , Mehdi N , de Cesar Netto C . Relationship between chronic lateral ankle instability and hindfoot Varus using weight‐bearing cone beam computed tomography. Foot Ankle Int. 2019;40(10):1175‐1181. 31253045 10.1177/1071100719858309

[10] Sugimoto K , Samoto N , Takakura Y , Tamai S . Varus tilt of the tibial plafond as a factor in chronic ligament instability of the ankle. Foot Ankle Int. 1997;18(7):402‐405. 9252808 10.1177/107110079701800705

[11] Scranton PE, Jr. , McDermott JE , Rogers JV . The relationship between chronic ankle instability and variations in mortise anatomy and impingement spurs. Foot Ankle Int. 2000;21(8):657‐664. 10966363 10.1177/107110070002100805

[12] Tümer N , Vuurberg G , Blankevoort L , Kerkhoffs G , Tuijthof G , Zadpoor AA . Typical shape differences in the subtalar joint bones between subjects with chronic ankle instability and controls. J Orthop Res. 2019;37(9):1892‐1902. 31042001 10.1002/jor.24336 PMC6772087

[13] Hayashi K , Tanaka Y , Kumai T , Sugimoto K , Takakura Y . Correlation of compensatory alignment of the subtalar joint to the progression of primary osteoarthritis of the ankle. Foot Ankle Int. 2008;29(4):400‐406. 18442455 10.3113/FAI.2008.0400

[14] Frigg A , Magerkurth O , Valderrabano V , Ledermann HP , Hintermann B . The effect of osseous ankle configuration on chronic ankle instability. Br J Sports Med. 2007;41(7):420‐424. 17261556 10.1136/bjsm.2006.032672 PMC2465368

[15] Probasco W , Haleem AM , Yu J , Sangeorzan BJ , Deland JT , Ellis SJ . Assessment of coronal plane subtalar joint alignment in peritalar subluxation via weight‐bearing multiplanar imaging. Foot Ankle Int. 2015;36(3):302‐309. 25380775 10.1177/1071100714557861

[16] Krähenbühl N , Tschuck M , Bolliger L , Hintermann B , Knupp M . Orientation of the subtalar joint: measurement and reliability using weightbearing CT scans. Foot Ankle Int. 2016;37(1):109‐114. 26293157 10.1177/1071100715600823

[17] Colin F , Horn Lang T , Zwicky L , Hintermann B , Knupp M . Subtalar joint configuration on weightbearing CT scan. Foot Ankle Int. 2014;35(10):1057‐1062. 25015393 10.1177/1071100714540890

[18] Kleipool RP , Dahmen J , Vuurberg G , et al. Study on the three‐dimensional orientation of the posterior facet of the subtalar joint using simulated weight‐bearing CT. J Orthop Res. 2019;37(1):197‐204. 30345548 10.1002/jor.24163

[19] Beimers L , Tuijthof GJ , Blankevoort L , Jonges R , Maas M , van Dijk CN . In‐vivo range of motion of the subtalar joint using computed tomography. J Biomech. 2008;41(7):1390‐1397. 18405904 10.1016/j.jbiomech.2008.02.020

[20] Kleipool RP , Natenstedt JJ , Streekstra GJ , et al. The mechanical functionality of the EXO‐L ankle brace: assessment with a 3‐dimensional computed tomography stress test. Am J Sports Med. 2016;44(1):171‐176. 26589838 10.1177/0363546515611878

[21] Dobbe JGG , de Roo MGA , Visschers JC , Strackee SD , Streekstra GJ . Evaluation of a quantitative method for Carpal motion analysis using clinical 3‐D and 4‐D CT protocols. IEEE Trans Med Imaging. 2019;38(4):1048‐1057. 30369440 10.1109/TMI.2018.2877503

[22] Colman KL , Dobbe JGG , Stull KE , et al. The geometrical precision of virtual bone models derived from clinical computed tomography data for forensic anthropology. Int J Legal Med. 2017;131(4):1155‐1163. 28185072 10.1007/s00414-017-1548-z PMC5491564

[23] Dukes KA . Gram–Schmidt process. *Wiley StatsRef: Statistics Reference Online*. American Cancer Society. 2014. https://onlinelibrary.wiley.com/doi/abs/10.1002/9781118445112.stat05633

[24] Cohen J . Statistical Power Analysis for the Behavioral Sciences. 2nd ed. Lawrence Erlbaum Associates; 1988.

[25] Terwee CB , Bot SD , de Boer MR , et al. Quality criteria were proposed for measurement properties of health status questionnaires. J Clin Epidemiol. 2007;60(1):34‐42. 17161752 10.1016/j.jclinepi.2006.03.012

[26] Colville MR . Surgical treatment of the unstable ankle. J Am Acad Orthop Surg. 1998;6(6):368‐377. 9826420 10.5435/00124635-199811000-00005

[27] Tourne Y , Mabit C . Lateral ligament reconstruction procedures for the ankle. Orthop Traumatol Surg Res. 2017;103(1S):S171‐S181. 27871968 10.1016/j.otsr.2016.06.026

[28] Peña Fernández M , Hoxha D , Chan O , et al. Centre of rotation of the human subtalar joint using weight‐bearing clinical computed tomography. Sci Rep. 2020;23;10(1):1035. 10.1038/s41598-020-57912-z PMC6978465 31974489

[29] Stufkens SA , Barg A , Bolliger L , Stucinskas J , Knupp M , Hintermann B . Measurement of the medial distal tibial angle. Foot Ankle Int. 2011;32(3):288‐293. 21477548 10.3113/FAI.2011.0288

[30] Cody EA , Williamson ER , Burket JC , Deland JT , Ellis SJ . Correlation of talar anatomy and subtalar joint alignment on weightbearing computed tomography with radiographic flatfoot parameters. Foot Ankle Int. 2016;37(8):874‐881. 27137795 10.1177/1071100716646629

